# Controllable Preparation of Spherical Molybdenum Nano-Powders by One-Step Reduction of APM in Radio Frequency Hydrogen Plasma

**DOI:** 10.3390/ma15062019

**Published:** 2022-03-09

**Authors:** Xiaoping Liu, Kuaishe Wang, Qiang Chen, Bing Zhang, Pengcheng Hao, Yuhao Wang, Qiang Wang

**Affiliations:** 1 School of Metallurgical Engineering, Xi’an University of Architecture and Technology, Xi’an 710055, China; qwc1228@163.com (Q.C.); bingzhang1112@xauat.edu.cn (B.Z.); honger@xauat.edu.cn (P.H.); wangyuhao@xauat.edu.cn (Y.W.); w18844216863@xauat.edu.cn (Q.W.); 2 National and Local Joint Engineering Center of Functional Materials, Xi’an 710055, China; 3 Key Laboratory of Gold and Resource of Shaanxi Province, School of Metallurgical Engineering, Xi’an University of Architecture and Technology, Xi’an 710055, China

**Keywords:** RF hydrogen plasma treatment, reduction, APM powder, nano-sized molybdenum powder

## Abstract

Spherical molybdenum nano-powders were in-situ ultrafast synthesized from ammonium paramolybdate (APM) raw materials in a one-step reduction method by radio frequency (RF) hydrogen plasma. Due to the extreme conditions of the RF plasma torch such as its high temperature and large temperature gradient, the injected raw APM powder was quickly gasified and then reduced into nano-sized metal molybdenum (Mo) powder. The influences of APM powder delivery rate and H_2_ concentration on the properties of the obtained powders were investigated. Field-emission scanning electron microscope (FESEM), transmission electron microscope (TEM), X-ray diffraction (XRD), nanolaser particle analyzer, and specific surface area method were used to characterize the morphology, phase, and particle size distribution of the powders. The results showed that the nano-sized Mo powder obtained by hydrogen plasma treatment had a quasi-spherical morphology and an average particle size of about 30 nm. The particle size could be successfully adjusted by varying H_2_ concentrations. In addition, spherical nano-sized MoO_3_ powder could be obtained when no H_2_ was added into the RF plasma.

## 1. Introduction

Because of its high melting points, good thermal conductivity, and superior corrosion resistance, as well as high yield strength and elastic modulus at high temperature, molybdenum and its alloys have become excellent materials for high-temperature structural parts in the fields of aerospace chemical, metallurgy, and nuclear [1,2]. Due to its high melting point of 2610 °C, molybdenum alloy components are generally prepared by powder metallurgy. The properties of molybdenum powder such as its particle size, purity, morphology, and dispersion have a great influence on the performances of molybdenum alloy components [3,4]. At present, the industrial production of high-quality molybdenum powder is always achieved through two-stage reduction of MoO_3_ (hydrogen reducing MoO_3_ to MoO_2_ at 600–700 °C, and further reducing MoO_2_ to Mo at 850–1100 °C). The molybdenum powders prepared by the hydrogen reduction method usually have large particle sizes of several microns because the molybdenum nucleation and growth process are difficult to control [5,6]. In addition, the sintering temperature of the micron-molybdenum powder must reach 1800–2000 °C and be maintained for several hours to obtain 90% of its theoretical density; the grain size of the compact is tens of microns [7,8,9]. Studies have shown that Mo nano-particles can be sintered at 1200 °C for one hour to prepare compacts over 95% of their theoretical density with an average grain size of 1.4 μm [10]. Alternatively, adding a small amount of nano-sized powder to the traditional micron-sized powder can also significantly reduce the sintering temperature of the traditional powder [9]. It can be seen that nano-molybdenum powder plays an important role in the sintering process of traditional parts. In addition, due to its size effect and activity, molybdenum nano-powders are often used as magnetic materials, metallurgical additives, chemical catalysts, etc. [11,12,13,14]. Mo nano-powders can be prepared by many methods such as high-energy ball milling, electric explosion, and other complex chemical reaction methods [15,16,17,18,19]. However, most of these methods require complicated procedures and cannot be used for industrial-scale mass production.

Over the last few decades, thermal plasma technology has attracted wide attention in synthesizing ultra-fine powders through gas-phase reaction due to its high temperature, high enthalpy, high quenching rate, clean reaction atmosphere, and wide range of controllable conditions [20,21,22,23,24,25]. Compared with DC plasma, radio frequency (RF) plasma has advantages such as a cleaner reaction atmosphere, higher energy, a larger reaction chamber, and being more suitable for synthesizing high-purity materials because of no electrode contamination. The spheroidization and refinement of many refractory metals and ceramic powders have been achieved using RF plasma, such as tantalum, tungsten, and AlO_3_ powders [26,27,28,29,30,31]. However, there are few reports on the preparation of spherical molybdenum powder by RF plasma, especially ultra-fine molybdenum powder [32]. In this paper, irregular ammonium paramolybdate (APM) powders under the carrying of powder delivery gas entered the hydrogen plasma flame (up to 8000 °C), underwent rapid cracking, reduction, and then were quenched to synthesize ultra-fine spherical metal molybdenum particles. Spherical molybdenum nano-powders were continuously prepared under dynamic hydrogen plasma in a one-step pathway from APM within a few seconds. The influences of H_2_ concentration and powder delivery rate on the obtained powder performance were examined. At the same time, the particle size of the as-obtained products was adjusted by changing the H_2_ concentration. The obtained rules can also be extended and applied to guide the preparation of other refractory metal and ceramic ultrafine spherical powders.

## 2. Materials and Methods

### 2.1. Experimental Equipment and Raw Materials

**Figure 1:**
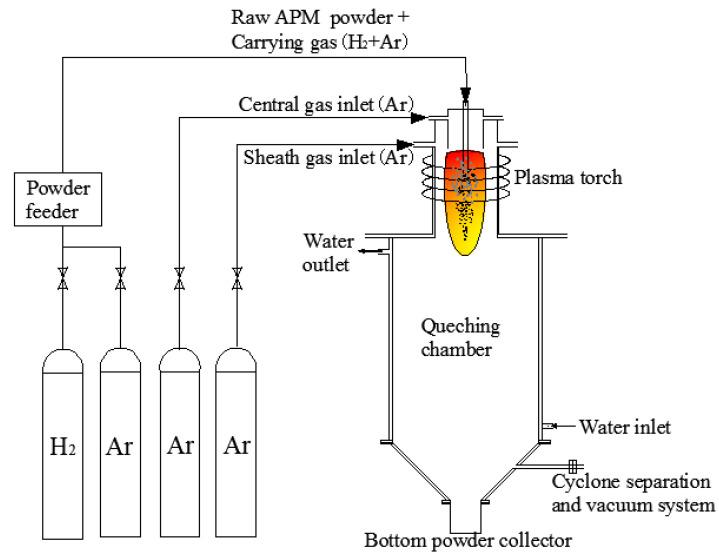
The schematic diagram of the equipment.

The preparation experiments of molybdenum nano-powder were carried out on RF plasma equipment. The experimental equipment included a radio frequency generator (36 kW, 4 MHz), a plasma generator, a double-layer stainless steel water-cooled chamber, and a bottom powder collector. The schematic diagram of the equipment is shown in Figure 1. The powder feeder is homemade, and its feed rate can be adjusted by the screw speed [33].

**Figure 2:**
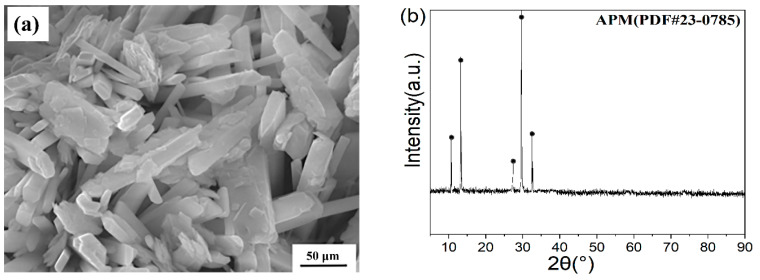
FESEM image (**a**) and XRD patterns (**b**) of the raw material APM powder.

The raw material powders of APM (ammonium paramolybdate, (NH_4_)_6_Mo_7_O_24_∙2H_2_O, 99.9%) were purchased from Jinduicheng Molybdenum Co., Ltd. (Xi’an, Shaanxi Province, China), in which the content of MoO_3_ was 81.55%. They are large white monoclinic crystals with a bulk density of 1.4 g/cm^3^, with almost no fluidity. The FESEM image and XRD pattern of the APM raw material are shown in Figure 2. It can be seen from Figure 2a that the APM precursor powders have a white flaky structure with particle sizes ranging from 10 µm to 50 µm. XRD patterns in Figure 2b indicate its good crystallization.

The hydrogen (H_2_) and argon (Ar) used in the experiment were both purchased from Xi’an Yatai Gas Company, with a purity of 99.9%.

### 2.2. Experiment Procedure

The plasma center gas and sheath gas used Argon (Ar, 99.9%). The powder-carrying gas was a mixture of argon and hydrogen (H_2_, 99.9%), and the ratio of hydrogen and argon can be adjusted according to the experiment’s needs. After the plasma torch heated the reaction chamber to a stable state for about 5 min, the dried APM precursor powders were delivered into the RF hydrogen plasma torch continuously using the mixture of Ar and H_2_ as the carrier gas. At the same time, hydrogen gas also played the role of reducing agent. APM powder underwent melting, evaporation, and reduction during the plasma processing. The resulting metallic molybdenum species condensed and formed spherical nano-particles with the help of the high quenching rate. Most of the as-obtained nano-sized powders fell freely to the bottom of the reaction chamber under the action of gravity to be collected. To maintain the stable operation of the plasma torch, it is crucial to set plasma parameters. The plasma parameter values in this work are arranged as shown in Table 1.

### 2.3. Characterization

The thermal decomposition of APM was analyzed by thermogravimetry (TG, Setaram Setsys Comprehensive Thermal Analyser in Franc). The phase composition of the raw powders and products was examined by X-ray diffractometer (XRD, D8 Advance A25, Bruker, Rheinstetten, Germany) in the 2θ-range from 20° to 90° with Cu Ka radiation (λ = 0.1540598) at a scanning speed of 4 deg·s^−1^. The morphology and microstructure of the particles were detected by field-emission scanning electron microscope (FESEM, Gemini 300, Oberkauchen, Germany) equipped with energy-dispersive X-ray spectroscopy (EDS), and a transmission electron microscope (TEM, Hitachi H-800, Tokyo, Japan). The chemical compositions of the product were measured by energy dispersive X-ray spectrometer (EDS, equipped on FESEM), Oxygen Nitrogen analyzers, and inductively coupled plasma atomic emission spectroscopy (ICP-AES, Optima 5300 DV, Shelton, WA, USA), tested three times under the same conditions, taking the average value as the final chemical composition value. The particle size of the powder was determined by high-resolution laser particle-size analyzer (LS13320, Beckman Coulter, Kraemer Boulevard, Brea, CA, USA), Nitrogen adsorption BET specific surface analyzer (ASAP-2020, Micromeritics, Norcross, GA, USA), and Nano measure statistical analysis software based on TEM.

The powder samples for both FESEM and TEM observation were ultrasonically dispersed for 15 min before testing. Then, the powder sample for FESEM was dried for 30 min, and three drops of the sample for TEM were dropped onto the carbon-coated copper mesh with a dropper and dried before the examination.

## 3. Results and Discussion

### 3.1. Preparation of the Molybdenum Nano-Powders by RF Hydrogen Plasma Reduction

**Figure 3:**
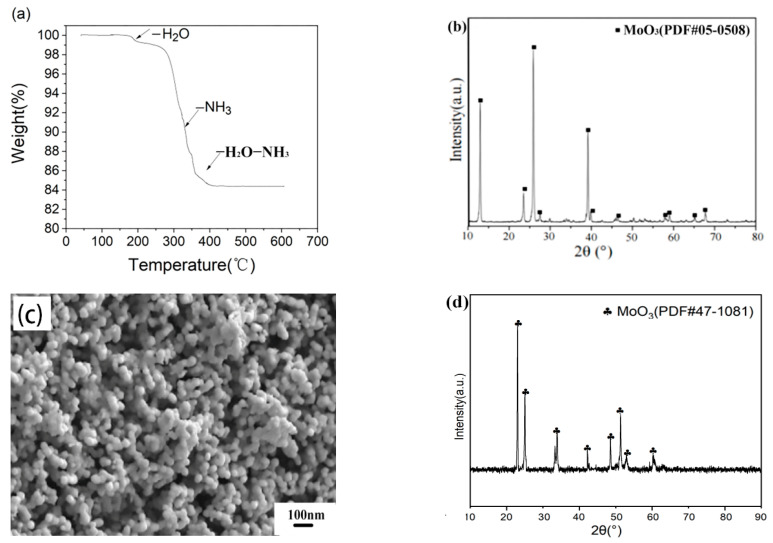
(**a**) TG analysis curve of APM decomposition in argon (heating rate 5 °C/min); (**b**) XRD patterns of resultant product in TG experiment; (**c**) FESEM image of obtained powders in an RF pure argon plasma without H_2_; (**d**) XRD patterns of the obtained product in a pure argon plasma.

Generally, the raw APM powder is easily decomposed when heated in the air, and the decomposition product is MoO_3_ [34]. In this work, the RF plasma used argon as the working gas. The thermogravimetric (TG) analysis of APM powder in the Ar atmosphere was carried out to study its decomposition process at high temperatures. The TG analysis curve of APM decomposition in argon (heating rate 5 °C/min) is shown in Figure 3a,b, i.e., the XRD patterns of the resultant product in the TG experiment. It was found that the initial decomposition temperature of APM powder in this TG experiment was 190 °C, and the whole decomposition included three steps: the loss of H_2_O (about 190 °C), NH_3_ (320 °C), and H_2_O-NH_3_ (390 °C), respectively. APM was completely decomposed into MoO_3_ near 400 °C. The TG results of APM in this work were almost consistent with those reported by Xiang Tiegen et al. [35]. Figure 3c,d shows the FESEM image and the XRD patterns of its decomposed product in an RF pure argon plasma torch. It can be seen that all peaks in the XRD patterns were indexed to the MoO_3_ structure when no H_2_ was added. The temperature of RF argon plasma (>3000 °C) can decompose almost arbitrary precursors. When APM powders were delivered into the high-temperature area of the argon plasma torch without adding H_2_ gas, it quickly lost H_2_O and NH_3_, cleaved, and decomposed into MoO_3_ fragments with an average particle size of about 30 nm, as shown in Figure 3c. This plasma treatment route without H_2_ addition can also be an effective method for preparing MoO_3_ nano-powders.

**Figure 4:**
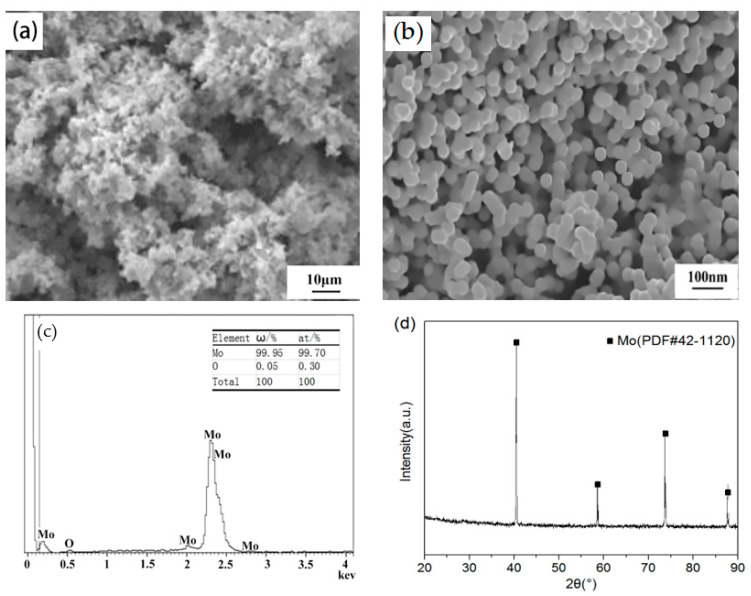
Characterization results of the obtained powders prepared by the hydrogen plasma at a delivery rate of APM: 30 g/min, H_2_ ratio: five times the stoichiometric level: (**a**) FESEM image with low magnification; (**b**) FESEM image with high magnification; (**c**) EDS plane scan spectrum of (**b**); (**d**) XRD pattern.

When a certain amount of H_2_ was added into the carrier Ar gas, nano-sized metal molybdenum particles could be obtained from APM in one-step RF plasma treatment because RF plasma provided a high concentration of active substances (H and H^+^ in this work) [28]. Figure 4 showed the morphology and structure of the resultant products when the APM powder delivery rate was 30 g/min and the ratio of H_2_ was five times the stoichiometric level. It can be seen from Figure 4a, combined with the manual visual inspection, that the product comprised black soot-like loose powders. In contrast, conventional micron-sized molybdenum powder is generally gray or dark gray. It is due to the small size effect of nano-sized powders, and the finer the powder particles, the darker the color. The FESEM image with high-magnification of the product in Figure 4b indicates that the obtained powders were quasi-spherical or an open-structure agglomerate composed of several quasi-spherical single particles. The chemical composition of the reduction product was evaluated with EDS, XRD, and Oxygen Nitrogen analyzers, respectively. Figure 4c is the EDS plane scan spectrum of area 2 in Figure 4b, indicating that the oxygen content of the reduced powder was extremely low, only 500 ppm, which approximately coincided with the result of 550 ppm measured by the oxygen and nitrogen analyzer. XRD patterns in Figure 4d reveal that the obtained powders were pure metal molybdenum without detectable oxides or other impurities. The difference from the EDS results is mainly attributed to the XRD accuracy of only 5%. The impurity contents in the powder before and after hydrogen plasma reduction measured by ICP-AES are shown in Table 2. It can be seen that the hydrogen plasma treatment greatly reduced the impurity content while reducing APM in one step, especially for low-melting-point elements. The reduced product powder was of high purity. Therefore, RF plasma also provides a way to purify substances.

**Figure 5:**
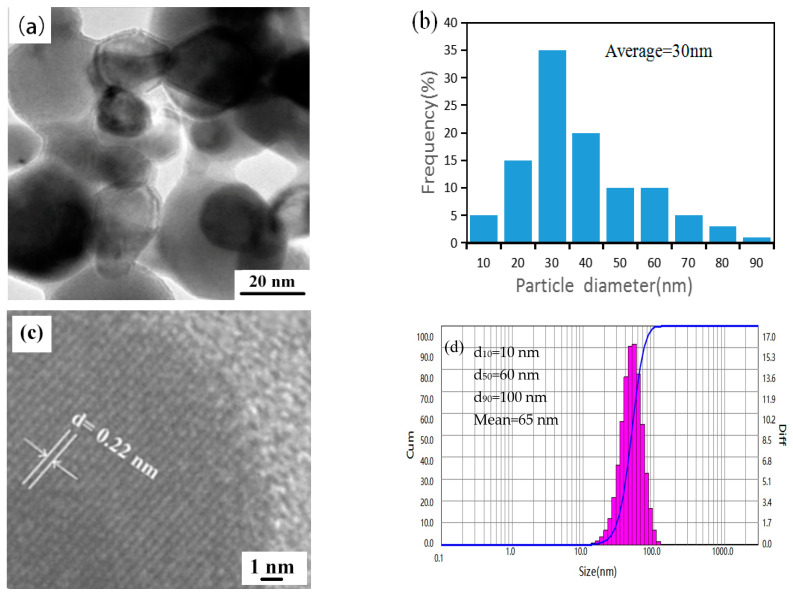
Characterization results of the obtained powders prepared by the hydrogen plasma at a delivery rate of APM: 30 g/min, H_2_ ratio: five times the stoichiometric level: (**a**) TEM micrograph; (**b**) particle-size distribution statistics based on TEM image; (**c**) HRTEM image; and (**d**) laser particle-size distribution curve.

TEM observation was carried out to confirm the microstructure of the reduction product. The TEM image in Figure 5a further demonstrates that the obtained powders had quasi-spherical morphology. HRTEM in Figure 5c further reveals the crystalline characteristics of a representative particle. The clarity of the crystal lattice fringes image indicates that the obtained powder had good crystallinity. The distance between adjacent lattice planes was 0.22 nm, which corresponds to the (110) crystal plane of Mo. Nano-measuring statistical analysis software equipped with TEM was used to calculate the particle size. The more particles measured in the statistical process, the more accurate the final statistical result. In this work, 100 particles with clear edges and uniform dispersion in TEM images were randomly selected as the measurement object. The statistical results are shown in Figure 5b, indicating that the particle size distribution of the reduction product was between 20 nm and 90 nm, and the particle size distribution was relatively narrow. At the same time, the average particle size of the obtained particles was only 30 nm. The laser particle size testing results in Figure 5d show that the average particle size was 65 nm, which is higher than that of the TEM method. It is because the laser particle-size testing method measures the particle size in a liquid medium, and agglomeration occurs between the product particles. At the same time, the product particles movement in the medium during the test will also cause the test results to be high. To further confirm the average particle size of the nano-powders, the specific surface area of the product was tested by a Nitrogen adsorption BET-specific surface analyzer at the same time. Assuming that the tested sample particles are rigid spheres, the average particle size of the product can be calculated by the formula $D=\frac{6}{A\rho }$ (where A is the specific surface area of the powder sample (m^2^/g), and ρ is the density of the sample (g/m^3^)). In this study, the test result of the specific surface area A of the product was 20.1 m^2^/g. The average particle size of the product nano-particles was calculated to be 32.4 nm, which was similar to the statistical result of 30 nm observed under TEM. Based on the above, excluding the influence of laser particle-size testing conditions, the particle size of the prepared nano-powder was about 30 nm.

The above studies show that when the powder feeding rate of APM was 30 g/min, the ratio of carrying gas H_2_ was five times the stoichiometric value, and that when RF plasma process parameters were set as in Table 1, spherical pure molybdenum nano-sized powders with a narrow and uniform powder particle size distribution with an average particle size of about 30 nm could be prepared.

**Figure 6:**
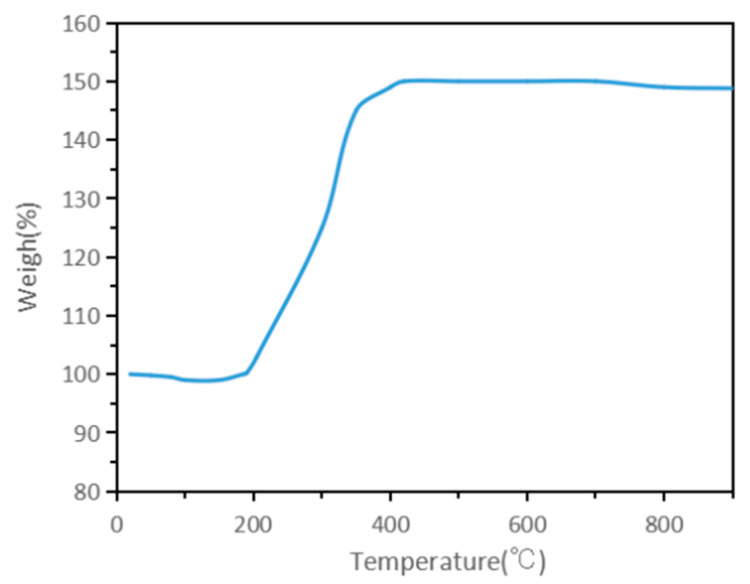
TG curve of the obtained molybdenum nano-sized powders (in air and a heating rate of 20 °C/min).

Conventional micron-sized molybdenum powder is stable in air and oxygen at room temperature, but its high-temperature oxidation resistance is very poor [36]. It started to oxidize when the ambient temperature reaches 400 °C, and rapidly oxidized into MoO_3_ at 500–600 °C. To comprehensively evaluate the oxidation resistance of the nano-sized molybdenum powders prepared by RF plasma in this study, thermogravimetric experiments((TG) were carried out under an air atmosphere. Figure 6 shows the thermogravimetric curve of the reduction product in an air atmosphere. It can be seen that the obtained nano-sized molybdenum powders were easily oxidized in an air atmosphere, and the oxidation reaction started at about 200 °C, which is about 200 °C lower than that of conventional molybdenum powders, and the oxidation speed was very fast. No significant change occurred after 400 °C, and all of them were oxidized to MoO_3_. It can be seen that with the further refinement of the particle size of molybdenum powder, its activity was enhanced and more easily oxidized. Therefore, when nano-sized molybdenum powders are prepared, it must be ensured that the RF plasma system used is strictly sealed. After the reaction, the equipment system should continue to be protected by inert gas to prevent the nano-sized molybdenum powders from oxidizing and spontaneously igniting due to contact with air. The collection chamber can be opened to take out the nano-molybdenum powder product until the entire system and the product are completely cooled. Similarly, when the prepared nano-sized molybdenum powder is heated or sintered, it must be isolated from air and oxygen at high temperatures. It can only be processed in a reducing atmosphere, an inert atmosphere, or an air atmosphere below 200 °C.

**Figure 7:**
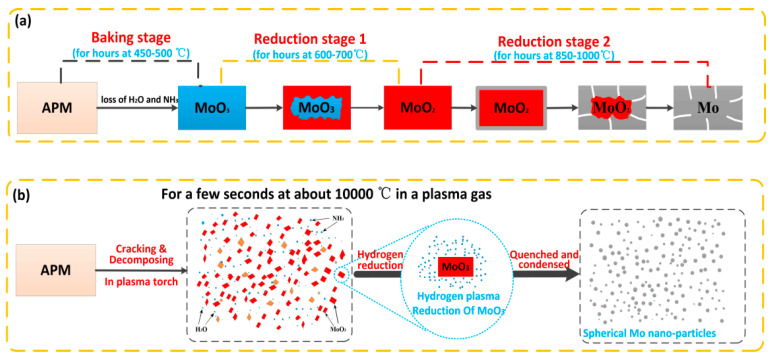
Schematic illustrations of the mechanism comparison to prepare molybdenum powder by APM hydrogen reduction: (**a**) traditional baking and two-stage reduction method, and (**b**) hydrogen plasma one-step reduction method.

Because of a series of properties such as extremely high temperature, excellent heat conductivity, strong reducibility, large reaction chamber, and rapid quenching rate, RF plasma can be used to synthesize metal Mo powders with nano-sized particles and spherical morphology. Figure 7 is a schematic illustration of the mechanism comparison between the plasma one-step reduction of APM to prepare nano-molybdenum powder and the traditional APM hydrogen reduction to prepare molybdenum powder. It can be seen that in the traditional hydrogen reduction method, the APM is first baked at a low temperature of 450–500 °C for several hours to obtain MoO_3_, and then the product MoO_3_ is reduced at 600–700 °C and 850–1000 °C, respectively, to obtain micron-sized molybdenum powder. As shown in Figure 7a, the reduction reaction is carried out from the outside to the inside in a static state, and the particle size and morphology are hereditary to a certain extent. Since the particle size of the raw material powder is in the micron scale, the product molybdenum powder can only be in the micron scale, rather than nanoscale. The whole calcination and reduction process are carried out in static state, each stage requires several hours, the preparation cycle is long, and the cost is high [37,38]. Compared with the traditional static reduction of MoO_3_ for several hours to prepare commercial Mo powder, it takes only a few seconds to prepare ultra-fine Mo powder by the hydrogen plasma-reducing APM in one step, which is a continuous dynamic process. Because the temperature of the plasma torch is as high as 3000 °C, when the APM raw powders are fed into the plasma flame, they are immediately decomposed and vaporized. The decomposition product of MoO_3_ undergoes a reduction reaction with the highly active hydrogen in plasma to generate metal molybdenum nuclei. Due to the high quenching rate in the plasma flame tail, the newly formed molybdenum nuclei do not have enough time to grow, agglomerate, and finally form ultra-fine metal molybdenum particles under the surface tension. During the transformation of APM into Mo, both physical phase transition and chemical reaction occur simultaneously. The equation for a possible reaction can be expressed as:

(NH_4_)_6_Mo_7_O_24_(s) = 2 MoO_3_(s) + 2 NH_3_(g) + H_2_O(g) (1)

MoO_3_(s) = MoO_3_(g) (2)

MoO_3_(g) + H_2_(g) = Mo(g) + H_2_O(g) (3)

Mo(g) = Mo(s)(4)

The reactions (1)–(3) occur in the heating section of the plasma torch, while the reaction (4) occurs in the quenching section. Reaction (1) is a fast thermal decomposition reaction in which APM is rapidly decomposed into MoO_3_ at the extremely high temperature of the plasma torch. After that, the solid MoO_3_ particles are evaporated into gaseous MoO_3_ under the action of high-temperature plasma, and reduced by hydrogen. Theoretically, the physical phase transition in reaction (2) and the reduction of MoO_3_ by hydrogen in reaction (3) compete and proceed simultaneously. The hydrogen reduction reaction of MoO_3_ can proceed spontaneously in both gaseous and solid MoO_3_. However, due to the slow-speed of the gas-solid reduction reaction between hydrogen and MoO_3_, which is carried out from the surface to the inside, and the rapid gasification of MoO_3_ being completed instantaneously by contrast, it can be deduced the chemical reaction should be carried out according to the gas-gas reduction reaction. The morphology change of the raw material before and after the reaction further proves that the reduction of APM in the plasma is a gas-gas reaction. This is because if it is a gas-solid reaction, the flake morphology of APM powders would be preserved instead of spherical. The reduction reaction with gaseous MoO_3_ takes only a few seconds in the plasma. Finally, the reduced product Mo vapor is cooled under the quenching condition of the tail of the plasma flame. The high quenching rate makes the nucleus grains have not enough time to grow and agglomerate, and form ultra-fine metal molybdenum particles under surface tension. The extremely high temperature of the hydrogen plasma torch and the extremely high quenching rate of the flame tail are the main reasons for the rapid reduction of APM and its retention of the products in the form of nano-molybdenum powder. It is worth noting that all the plasma processes are completed in flowing gas in a continuous way in a few seconds, indicating the effectiveness of the synthetic route, which can be easily carried out on a large scale without pollution.

RF hydrogen plasma provides an effective way to produce nano-sized molybdenum powders in a one-step way, and the method is also promising to be extended to other refractory metals. In particular, two factors that have an influence on the formation of Mo nano-sized powders were investigated as follows.

### 3.2. Influence of H_2_ Concentration

**Figure 8:**
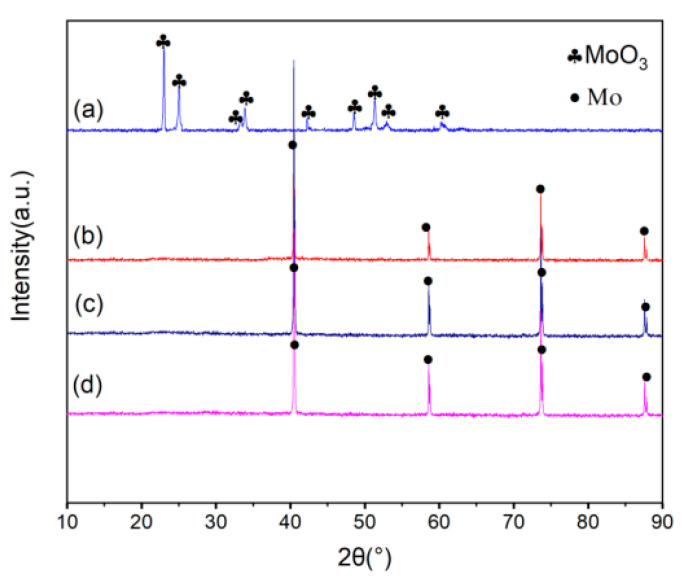
XRD of the product powders at different H_2_/stoichiometric H_2_: (**a**) 0, (**b**) 1.5, (**c**) 3.0, (**d**) 5.0.

**Figure 9:**
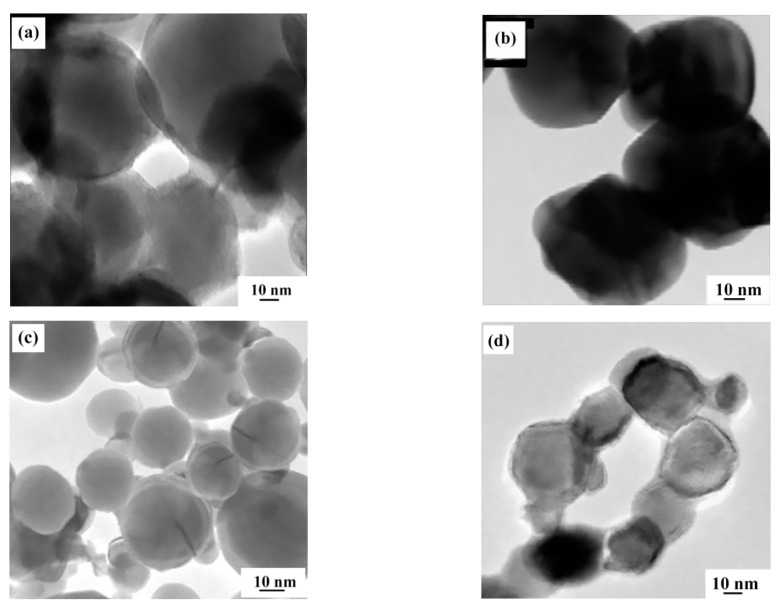
TEM images of the obtained ultrafine Mo powders at different ratios of H_2_/stoichiometric H_2_: (**a**) 0, (**b**) 1.5, (**c**) 3.0, (**d**) 5.0.

**Figure 10:**
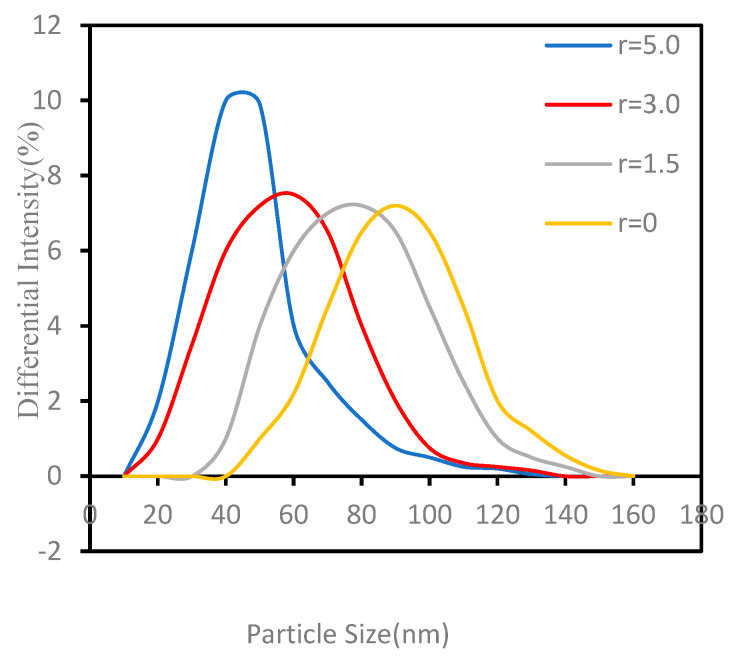
Laser particle−size distributions of the obtained ultrafine Mo powders at different r: H_2_/stoichiometric H_2_.

Experiments were performed by changing the ratio of H_2_ to H_2_ needed to reduce APM (that is, the stoichiometric level) for exploring the influence of H_2_ concentration. The delivery rate of APM was set at 30 g/min, and the other experimental parameters were selected as the values in Table 1. The products consisted of only the MoO_3_ phase when APM powder was injected into pure Ar plasma flame without H_2_. When the added H_2_ concentration exceeded the stoichiometric level, the MoO_3_ disappeared, and the product became pure metal Mo at last, as shown in Figure 8. The TEM images of the Mo nano-powders with different H_2_ concentrations in Figure 9 reveal that the particle size of the prepared nano-powder decreased with the increase of H_2_ concentration. When no hydrogen was added to the Ar plasma, spherical MoO_3_ particles with an average particle size of 90 nm could be obtained. The average size of the product Mo powders was about 70 nm when the H_2_ concentration was 1.5 times the stoichiometric value and reduced to 30 nm as the ratio raised to 5.0. This variation can also be further verified by the laser particle-size testing results in Figure 10. With the increment of H_2_ ratio, the concentration of active H^+^ and H in the RF plasma torch increased, contributing to the increment of plasma torch temperature, and the APM precursor powders can be broken into smaller fragments which were reduced into smaller nano-sized metal Mo powder. Therefore, the particle size of the obtained Mo nano-powders can be controlled by adjusting the ratio of H_2_ to stoichiometric H_2_. In addition, pure Ar RF plasma is also a new way to prepare MoO_3_ nano-powders using APM as a raw material directly.

### 3.3. Influence of the Raw APM Powder Delivery Rate

**Figure 11:**
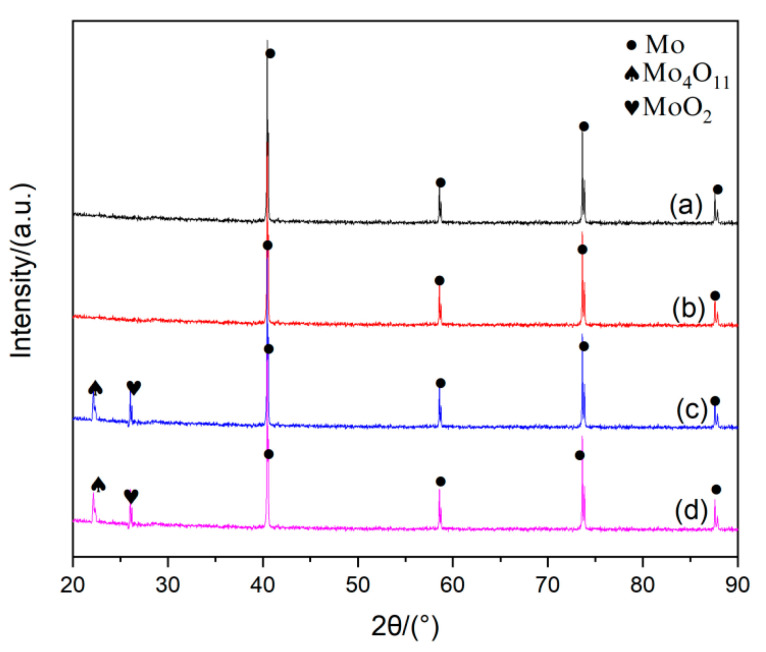
XRD patterns of the obtained powders under different APM powder delivery rates: (**a**) 30 g/min; (**b**) 40 g/min; (**c**) 50 g/min; (**d**) 60 g/min.

Experiments were carried out to determine the influence of the APM powder delivery rate on the formation of Mo nano-powders through changing the feeding weight of APM powder per minute (in which the ratio of H_2_ to stoichiometric H_2_ was 1.0, the other experimental parameter were set as the values in Table 1). When the precursor powder APM was delivered dispersedly into the RF plasma torch, the gray and loose nano-sized metal Mo powders were prepared, and the feeding rate had an influence on their chemical compositions, as shown in Figure 11. The purity of metal Mo ultra-fine powders can be adjusted by the raw APM powder delivery rate. When APM was delivered into the high-temperature plasma torch at the delivery rate of 30 g/min or 40 g/min, as shown in the XRD pattern in Figure 11a,b, the product powder was composed of only metal molybdenum phase. No impurities such as molybdenum oxide were detected. While, as the APM delivery rate was increased to 50 g/min or more, as shown in Figure 11c,d, there were some molybdenum oxides (Mo_4_O_11_ and MoO_2_) included in the product molybdenum powders. The reduction of APM is an endothermic process. When the amount of APM powder added to the plasma flame is excessive, the fed powder does not have enough energy to absorb. The reaction cannot be entirely carried out, resulting in the decreasing of the purity. Therefore, to ensure the purity of the obtained nano-sized Mo powder, the powder delivery rate must be controlled within the appropriate range.

## 4. Conclusions

In the present study, a new RF hydrogen plasma reduction method was proposed for the ultra-fast synthesis of spherical nano-sized molybdenum powders. Then, the micromorphology, chemical composition, and particle size distribution of the reduction products were explored. In addition, the effects of H_2_ concentration and powder feeding rate were examined. The following conclusions can be drawn:

(1) Pure metallic Mo nano-powders with spherical morphology can be ultra-fast prepared in a one-step way by hydrogen plasma reduction in a few seconds using APM powder as the precursor.
(2) The average size of the obtained Mo powder was about 30 nm with uniform particle size distribution and narrow range.
(3) The phase compositions of nano-sized powders can be adjusted by changing the delivery rate of the APM precursor, and the particle size of the synthesized Mo nano-powders can be adjusted through varying H_2_ concentrations.
(4) RF hydrogen plasma develops a straightforward pathway to prepare Mo nano-powders from raw APM powders on a large scale. In addition, pure Ar plasma without H_2_ also provides a new way to prepare high-purity MoO_3_ nano-powders directly from APM precursors.

## Figures and Tables

**Table 1:** The values of experimental parameters for APM reduction by the RF plasma.

Parameter	Value (25 °C, 86.1 kPa)
Plasma power	30 kW
Central gas (Ar)	15 L/min
Sheath gas (Ar)	45 L/min
Carrier gas (H_2_ + Ar)	8 L/min
Powder delivery rate	30–60 g/min

**Table 2:** Composition results of the ICP-AES analysis of the powders before and after RF treatment (μg/g).

Element	Fe	Si	Mn	Al	Mg	Ni	Ti	V	Co	P	Sb	Cu	Cr	W
Raw APM powder	<8	<8	<4	<6	<6	<3	<10	<10	<4	<5	<5	<3	<10	<100
Reduced powder	<4	<2	<3	<0	<0	<1	<4	<6	<3	<3	<3	<1	<6	<98

## Data Availability

The data presented in this study are available on request from the corresponding author.
